# Recommendations of the clinical target volume for the para-aortic region based on the patterns of lymph node metastasis in patients with biliary tract cancer

**DOI:** 10.3389/fonc.2022.893509

**Published:** 2022-11-03

**Authors:** Xin Long, Han Wu, Lei Yang, Hui Xu, Jing Dai, Wenbo Wang, Ling Xia, Jin Peng, Fuxiang Zhou

**Affiliations:** Department of Radiation and Medical Oncology, Hubei Key Laboratory of Tumor Biological Behaviors, Hubei Cancer Clinical Study Center, Zhongnan Hospital of Wuhan University, Wuhan, China

**Keywords:** biliary tract cancer, lymph node metastases, clinical target volume, adjuvant radiotherapy, para aortic region

## Abstract

**Background:**

Even though the clinical target volume (CTV) in biliary tract cancer (BTC) patients has been proposed by several previous studies, the para aortic CTV for BTC is still not well-defined. The objective of this study was to determine the precise delineation of the para aortic CTV for BTC according to the distribution pattern and failure pattern of lymph nodes.

**Methods:**

Computed tomography (CT)-, magnetic resonance imaging (MRI)- or positron emission tomography-computed tomography (PET-CT)-generated images of patients with BTC from 2015 to 2020 were analyzed retrospectively. The distribution patterns of lymph nodes in different regions were summarized. The diagnosed para aortic lymph nodes (PALNs) were manually mapped to standard axial CT images. The asymmetric CTV expansions from the para aortic were defined according to the distance from the volumetric centre of lymph node to the most proximal border of aorta.

**Results:**

A total of 251 positive lymph nodes were found in the study cohort (n = 61 patients, 92 PALN). All PALNs were projected onto axial CT image of the standard patient. PALNs were concentrated in the 16a2 and 16b1 regions, and the involvement rates were 17% and 13% respectively. Therefore, the upper boundary of 16a2 and the lower boundary of 16b1 were defined as the cranial and caudal border of para aortic CTV, respectively. For the study cohort, the mean distance from the volume center of all lymph nodes in 16a2 and 16b1 to the proximal border of the aorta was 9 mm (range 4-24) in the front, 7 mm (range 3-14) on the left, and 12 mm (range 5-29) on the right. For the validation cohort (n=19 patients, 56 PALN), the mean distance from the center of the lymph node to the border of the aorta were both 10 mm on the left (range 5-20) and right (range 6-23). The mean distance in front of the aorta was 9 mm (range 5–23). Finally, a CTV expansion from the aorta of 18 mm in the front, 12 mm on the left, and 24 mm on the right resulted in 96% (73/76) coverage of PALNs in the study cohort. At the time of the validation, the described CTV could include 96% (47/49) of recurrent PALNs in the validation cohort.

**Conclusions:**

The involvement rates of PALNs in 16a2 and 16b1 were the highest. Based on the distribution of PALNs, a new para-aortic CTV was defined to construct a more accurate target volume for adjuvant radiotherapy in BTC.

## 1 Introduction

Biliary tract cancer (BTC) refers to a spectrum of invasive adenocarcinomas, including cholangiocarcinoma and gallbladder carcinoma (GBC) (1). The prognosis is poor, with a 5-year survival of approximately 5%–15% (all stages jointly analyzed) (2, 3). Complete surgical resection is the cornerstone of treatment (4), but after surgical resection, relapse rates are high, with approximately 60%–70% of patients expected to have disease recurrence (5). The local recurrence rate ranges from 39% to 69% (6). The trend of local recurrence of BTC provides a rationale for additional adjuvant therapy after definitive surgery.

The phase II clinical study SWOGs 0809 (6) demonstrated that adjuvant radiotherapy for BTC is beneficial to local control and survival. The NCCN guidelines recommend that patients with positive resection margins or positive lymph nodes should receive postoperative adjuvant radiotherapy. Usually, the delineation of the clinical target volume (CTV) of adjuvant radiotherapy is based on the pattern of regional lymph node metastasis (LNM). According to the AJCC cancer staging manual, diseases that spread to the celiac, periaortic, or caval lymph nodes were usually classified as M1 in the TNM classification of BTCs. However, clinical radiation fields generally encompass the periaortic or caval lymph nodes. A meta-analysis (7) showed that the common lymph node recurrence areas of intrahepatic cholangiocarcinoma (iCCA), extrahepatic cholangiocarcinoma (eCCA), and GBC included the para-aortic, with involvement rates of 7%, 15.2%, and 6%, respectively. A review article by Socha et al. (8) assessed the risk of lymph node involvement based on histopathological surgical data and the areas of inclusion in the target volume of adjuvant radiotherapy in BTC and found that the para-aortic region was a high-risk and potential missed area for any tumor sites in the biliary tract. Because of the high metastasis rate of the retroperitoneal lymph node and the low surgical dissection rate (9–11), adjuvant radiotherapy is preferred to eliminate potential microscopic disease within the clinical irradiation field. However, there is still a lack of an accurate and reproducible para-aortic CTV recommendation in BTC. Inconsistent delineation standards of adjuvant radiotherapy between different treatment institutions may lead to poor local control in small para-aortic CTV settings or serious adverse reactions in large para-aortic treatment fields.

Therefore, the purpose of this study was to identify high-risk lymphatic drainage regions according to the pattern of LNM and construct a more accurate para-aortic CTV according to the distribution pattern of para-aortic lymph nodes (PALNs) to provide a reference for the CTV delineation of adjuvant radiotherapy in malignant biliary tumors.

## 2 Methods

### 2.1 Patients

#### 2.1.1 Study cohort

This study was approved by the internal Institutional Review Board of our hospital (IRB number 2021007K). Between January 2015 and July 2020, the medical charts of BTC patients with LNM who were diagnosed at the first examination were reviewed and selected as the study cohort. The detailed inclusion and exclusion criteria are shown in **Supplementary Figure 1A**. Furthermore, the flowchart of the study cohort is shown in **Supplementary Figure 1B**.

#### 2.2.2 Validation cohort

During the same period, BTC patients with postoperative recurrence were selected, among whom BTC patients with lymph node recurrence diagnosed by imaging were identified as the validation cohort. The patient selection criteria and the study design flowchart are shown in **Supplementary Figure 2A**. Furthermore, the flowchart for the validation cohort is shown in **Supplementary Figure 2B**.

### 2.2 Evaluation of LNM

The imaging diagnostic methods of LNM include computed tomography (CT), magnetic resonance imaging (MRI), or positron emission tomography–computed tomography (PET-CT). The positive lymph nodes on images were identified by the following features (1): the lymph nodes were round in shape with a short axis length ≥1 cm or a ratio of length to diameter of <2; (2) there was an infiltrating margin; (3) the lymph nodes were increased in number and size compared with previous images; (4) central necrosis or non-uniform enhancement was present; (5) responsiveness to treatment; and (6) the standard uptake value (SUV) was >2.5 on PET-CT (12). LNM needs to be evaluated by two experienced radiologists. No histological confirmation of LNM was obtained.

### 2.3 Metastatic lymph node classification and mapping

The classification of lymph nodes is based on the anatomical definition of lymph node stations in the Japanese Gastric Cancer Association (JGCA) (13). A patient without LNM in our center was selected as the standard patient. This patient underwent a planned CT scan in the supine position. The scan margins were from the top of the skull to the level of the L5 vertebra, and the slice thickness was 3 mm. Two radiation oncologists and one radiologist mark the LNM locations onto the corresponding anatomic positions in the axial CT images of the standard patient, with reference to the surrounding vascular and bony structures (14). All nodes are replaced by points with a diameter of 6 mm. The lymph nodes were plotted in the Varian planning system (version 13.5; Varian Medical Systems, Palo Alto, CA, USA).

### 2.4 Design of the para-aortic CTV

First, based on the involvement rate of the para-aortic region, the cranial and caudal borders of the para-aortic CTV were determined. Then, all PALNs were classified as left para-aortic (LPA) and right para-aortic (RPA) or anterior para-aortic (APA) and posterior para-aortic (PPA) based on their location. The expansion margins from the aorta were determined by the distance from the border of the aorta to the center of all lymph nodes between the cranial and caudal borders of the CTV. Initially, the margins were expanded by the mean distances from the aorta to the lymph node center. Expansion margins were then asymmetrically increased, based on the PALN distribution, until a clinically acceptable lymph node coverage was obtained. For these iterations, the 90th and 95th percentiles of the distances from the lymph node center to the aorta were calculated. These expansions were combined to create a CTV. The most suitable combination could cover more than 95% lymph nodes (15). The CTV avoided the bowel, muscle, and bone.

### 2.5 Validation of the para-aortic CTV

The PALNs in the validation cohort were also projected on the axial CT images of the standard patient. The expansion margins calculated from the study cohort were applied, and the coverage of the lymph node center in the validation cohort was assessed. A total coverage of ≥95% is considered reasonable and acceptable.

### 2.6 Statistical analysis

All data are presented as medians with interquartile ranges (IQRs) for continuous variables and numbers with percentages for categorical variables. Survival analysis was estimated by the Kaplan−Meier method, and the impact of different recurrence sites on survival was tested with the log-rank test. Overall survival (OS) was calculated from the time of surgery until the date of death or the latest follow-up. All statistical analyses were performed using SPSS version 26.0. All graphs were processed using the commercially available photo-editing software Adobe Photoshop CC 2019.

## 3 Results

### 3.1 Patient characteristics in the study cohort

A total of 218 patients diagnosed with BTCs from January 2015 to July 2020 in our hospital were enrolled. Sixty-one newly diagnosed patients with LNM were identified as the study cohort, including 15 patients with GBC, 35 patients with iCCA, and 11 patients with eCCA. There were 31 patients with PALNs in the study cohort (**Table 1**).

**Table 1:** Characteristics of patients in the study cohort.

Variable	Study cohort N = 61
Age (y), median (IQR)	60 (52-66)
Sex	
Men	33 (54%)
Women	28 (46%)
Tumor location	
Gallbladder	15 (25%)
Intrahepatic bile duct	35 (57%)
Extrahepatic bile duct	11 (18%)
Histology	
Adenocarcinoma	57 (93%)
Other types	4 (7%)
PALN	
With PALN	31 (51%)
Without PALN	30 (49%)
Number of PALNs	92

IQR, interquartile range; iCCA, intrahepatic cholangiocarcinoma; PALN, para-aortic lymph node.

### 3.2 Distribution of positive lymph nodes in the study cohort

**Figure 1:**
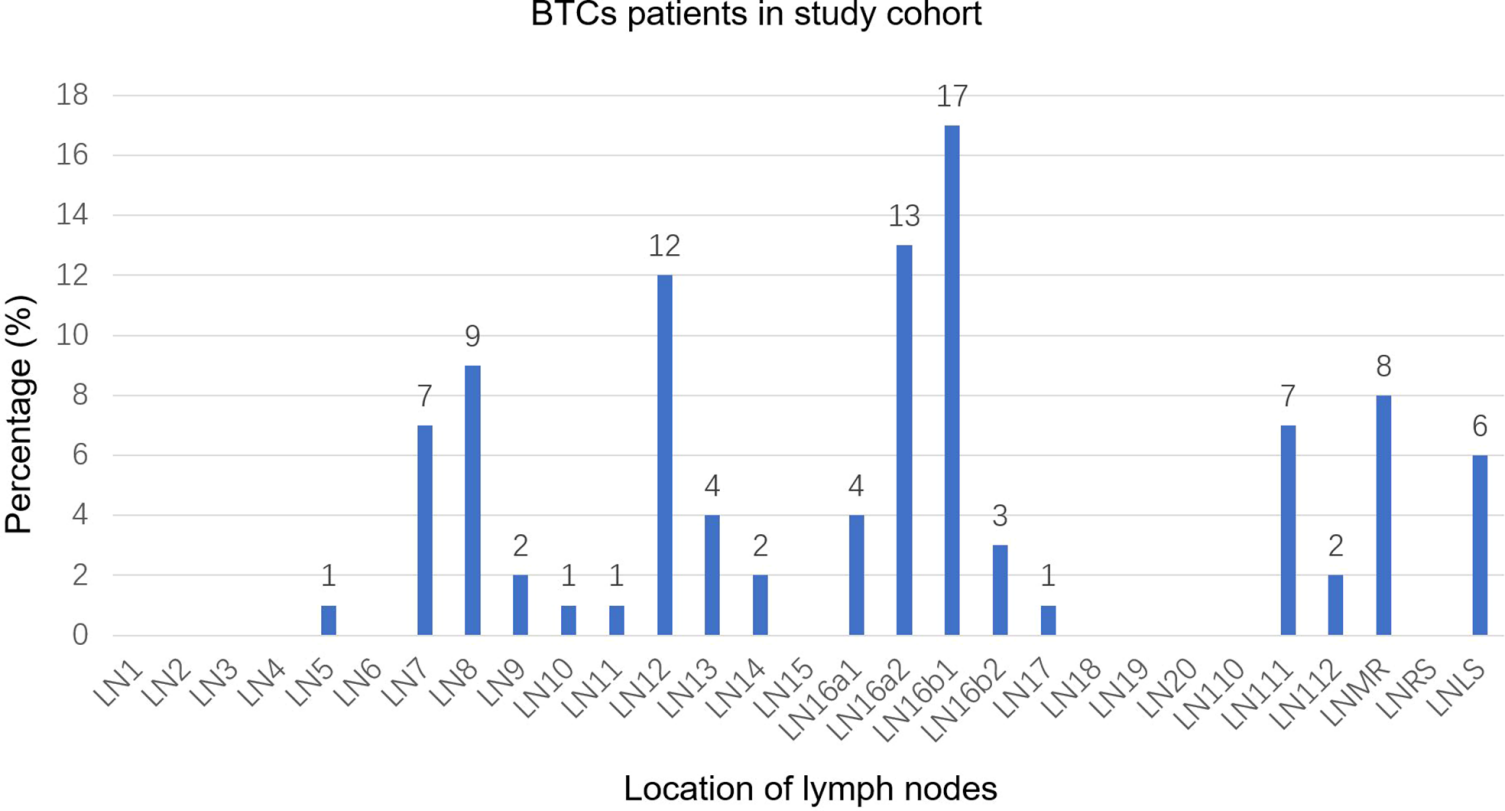
Distribution bar plot of positive lymph nodes in the study cohort. BTCs, biliary tract cancer; LN, lymph node; LNMR, mediastinal lymph node; LNRS, right supraclavicular lymph node; LNLS, left supraclavicular lymph node.

A total of 251 lymph nodes were identified in the study cohort. These lymph nodes were mainly concentrated in regions 16a2 (13%) and 16b1 (17%), followed by regions 12 (12%) and 8 (9%). Regions 9, 10, 11, 13, 14, 16a1, 16b2, 17, and 112 showed lower involvement rates. Other regions, including region 7, region 111, the mediastinal region, and the left supraclavicular region, showed moderate involvement rates of the lymph node (**Figure 1**). According to the different tumor locations, results of the subgroup analysis showed that region 16b1 was the most common area of LNM for both iCCA and GBC, with involvement rates of 17% and 18%, respectively, followed by region 16a2 (11%), region 111 (11%), and the mediastinal region (11%) in iCCA and the left supraclavicular region (17%) and region 12 (15%) in GBC (**Supplementary Figures 3A–B**). In eCCA, the highest involvement region was 16a2 (28%), followed by regions 12 (24%) and 16b1 (17%) (**Supplementary Figure 3C**). There were 92 PALNs, and over half (60%) of the PALNs were located in the LPA, 40% were located in the RPA, and 60% were located in the APA (**Supplementary Figure 4**).

### 3.3 Delineation of the para-aortic CTV in the study cohort

**Figure 2:**
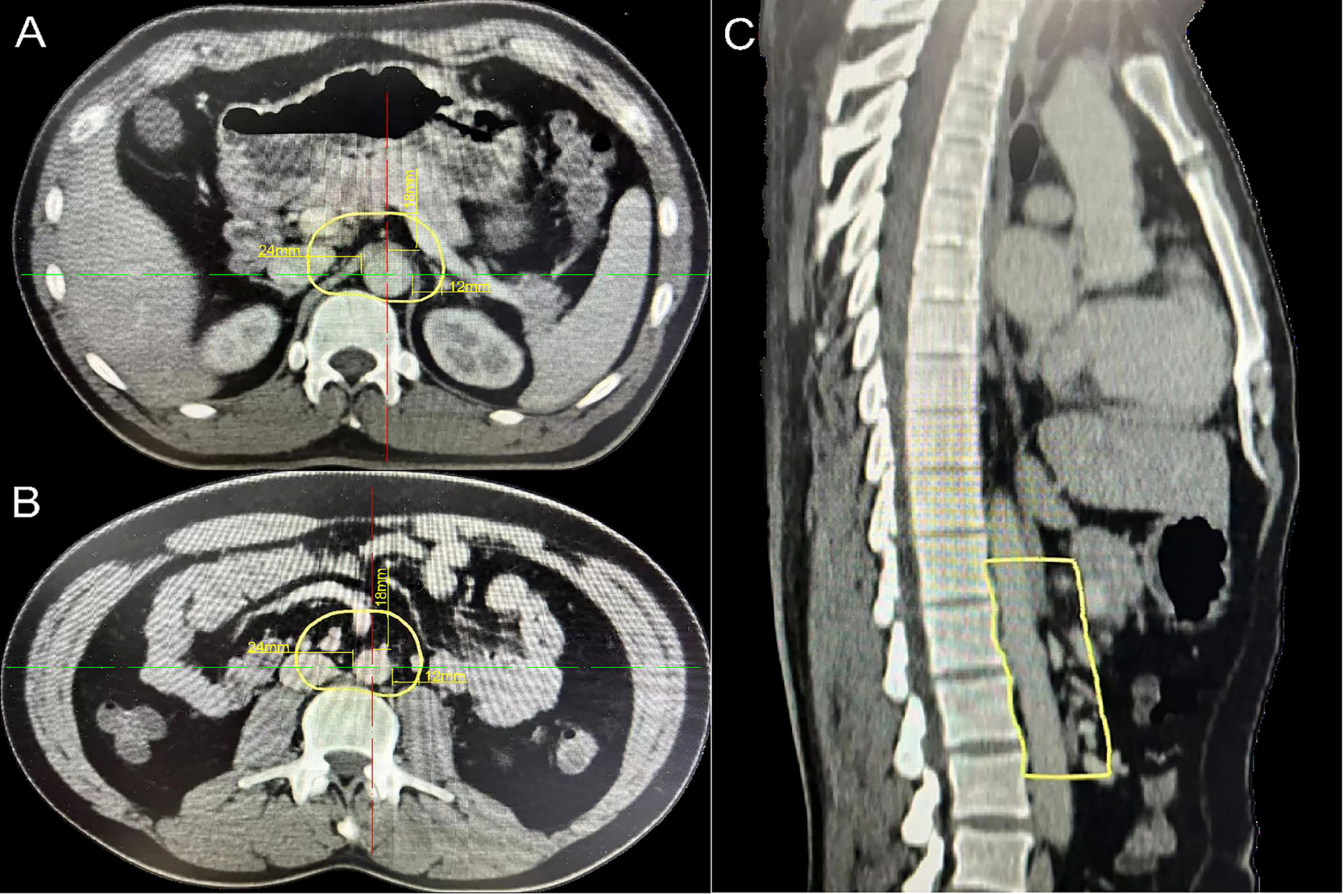
Proposed atlas of the para-aortic CTV in the study cohort. **(A)** The cranial border of the para-aortic CTV (aorta expansion with 18 mm in the front, 12 mm on the left, and 24 mm on the right). **(B)** The caudal border of the para-aortic CTV (aorta expansion with 18 mm in the front, 12 mm on the left, and 24 mm on the right). **(C)** Para-aortic CTV (sagittal plane). CTV, clinical target volume.

A total of 92 PALNs in the study cohort were projected onto the axial CT images of standard patients. In the para-aortic region, 16a2 and 16b1 had extremely high involvement rates (**Supplementary Figures 5A–D**). Therefore, the upper boundary of the 16a2 region, that is, the superior border of the celiac trunk, was defined as the cranial border of the para-aortic CTV, and the lower boundary of the 16b1 region, that is, the superior border of the inferior mesenteric artery, was defined as the caudal border of the para-aortic CTV, which is equivalent to the middle level of the T12 vertebra to the superior to the L3 vertebra. In the horizontal direction, the mean distances from the volumetric center of all lymph nodes in 16a2 and 16b1 to the proximal border of the aorta were 9 mm (range 4–24) for APA, 7 mm (range 3–14) for LPA, and 12 mm (range 5–29) for RPA. The 90th percentile distances from the center of the lymph nodes to the border of the aorta were 14 mm for APA, 11 mm for LPA, and 22 mm for RPA. The 95th percentile distances were 18, 12, and 24 mm for APA, LPA, and RPA, respectively (**Table 2**). The expansion margins from the aorta at 9 mm in the front, 7 mm on the left, and 12 mm on the right received 67% (n = 51/76) coverage of the lymph nodes. The expansion margins from the aorta at 14 mm in the front, 11 mm on the left, and 22 mm on the right received 93% (n = 71/76) coverage. The expansion margins from the aorta at 18 mm in the front, 12 mm on the left, and 24 mm on the right received 96% (n = 73/76) coverage. The margins were cropped at the bowel, muscle, and bone (**Figures 2A–C**; **Supplementary Figure 6**).

**Table 2:** Distance from the aorta to the volumetric center of the PALN in LN16a2 and LN16b1 in the study cohort.

Location of PALNs	Mean distance from the aorta (mm) (range)	The 90th percentile of the distances (mm)	The 95th percentile of the distances (mm)
LPA	7 (3–14)	11	12
RPA	12 (5–29)	22	24
APA	9 (4–24)	14	18

PALN, para-aortic lymph node; LPA, left para-aortic; RPA, right para-aortic; APA, anterior para-aortic.

### 3.4 Patient characteristics in the validation cohort

Forty-five patients with postoperative recurrence were enrolled, including 12 GBC, 18 iCCA, and 15 eCCA patients. Among them, 21 patients had intrahepatic recurrence only, seven patients had distant organ recurrence (peritoneum, bone, and muscle), and 19 patients had lymph node recurrence (**Supplementary Table 1**). Nineteen patients with lymph node recurrence were selected as the validation cohort (**Table 3**). There were 14 patients with PALN recurrence in the validation cohort.

**Table 3:** Characteristics of patients in the validation cohort.

Variable	Validation cohort (N = 19)
Age (y), median (IQR)	57 (48-64)
Sex	
Men	12 (63%)
Women	7 (37%)
Tumor location	
Gallbladder	4 (21%)
Intrahepatic bile duct	9 (47%)
Extrahepatic bile duct	6 (32%)
Approach of operation	
Radical resection of hilar cholangiocarcinoma	2 (11%)
Radical pancreaticoduodenectomy	4 (21%)
Radical cholecystectomy	4 (21%)
Resection of iCCA	9 (47%)
Postoperative adjuvant therapy	
Chemotherapy	1 (5%)
Radiotherapy	1 (5%)
Concurrent chemoradiotherapy	3 (16%)
No adjuvant therapy	14 (74%)
Histology	
Adenocarcinoma	19 (100%)
Other types	0 (0%)
PALN	
With PALN	14 (74%)
Without PALN	5 (26%)
Number of PALNs	56

IQR, interquartile range; iCCA, intrahepatic cholangiocarcinoma; PALN, para-aortic lymph node.

### 3.5 Distribution of positive lymph nodes in the validation cohort

**Figure 3:**
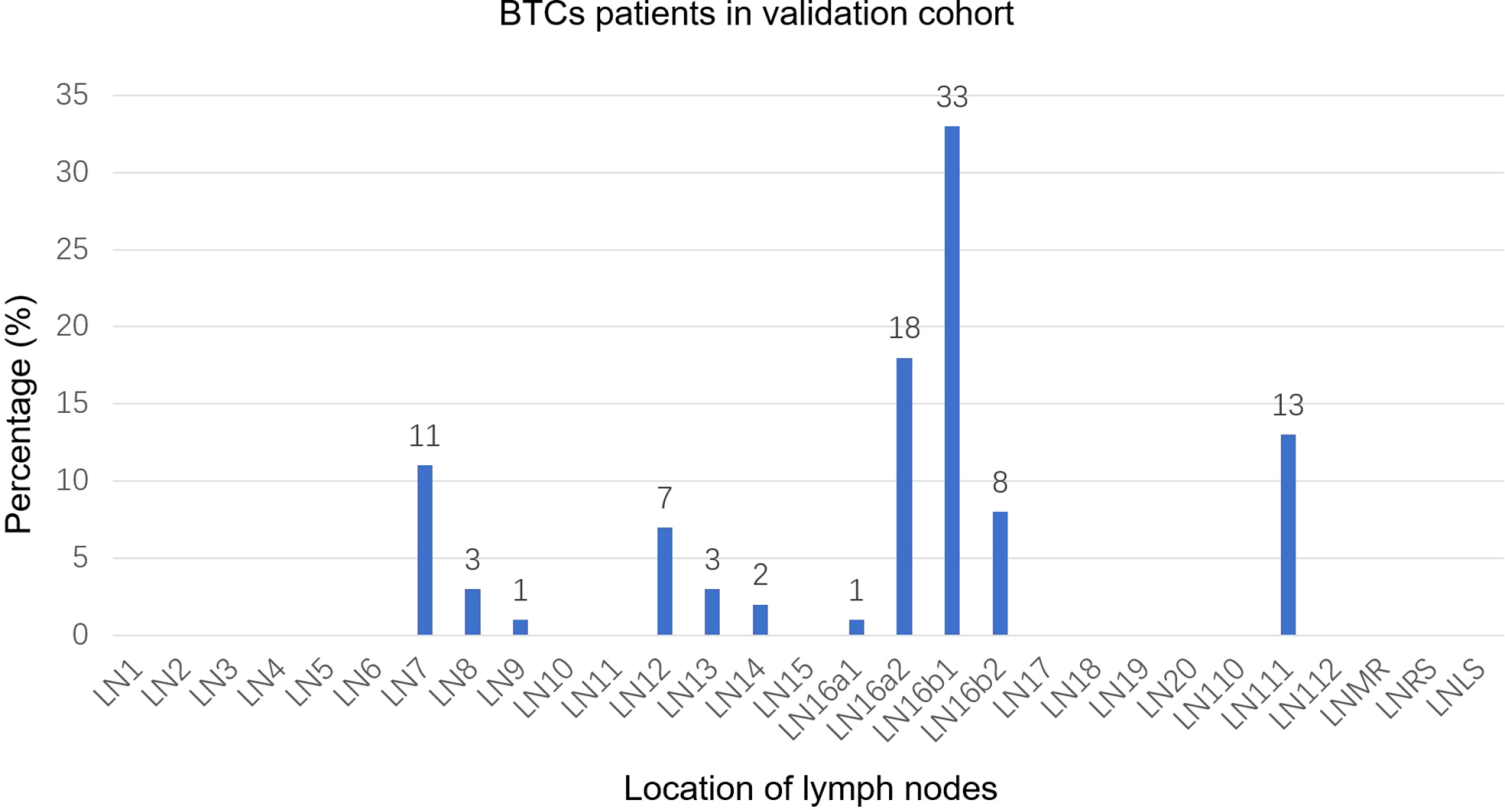
Distribution bar plot of positive lymph nodes in the validation cohort. BTCs, biliary tract cancer; LN, lymph node; LNMR, mediastinal lymph node; LNRS, right supraclavicular lymph node; LNLS, left supraclavicular lymph node.

A total of 93 positive lymph nodes were diagnosed in the validation cohort. These lymph nodes are mainly distributed in regions 16b1 (33%) and 16a2 (18%). The involvement rates of regions 111 and 7 were 13% and 11%, respectively (**Figure 3**). The involvement rates of region 16b1 in GBC, iCCA, and eCCA were 29%, 37%, and 32%, whereas the involvement rates of region 16a2 were 7%, 26%, and 8%, respectively (**Supplementary Figures 7A–C**). There were 56 PALNs in this cohort, and the distribution of PALNs was similar to that in the study cohort. Approximately 70% of the lymph nodes were located in LPA, 30% in RPA, and 59% in APA (**Supplementary Figure 8**).

### 3.6 Para-aortic CTV coverage in the validation cohort

**Figure 4:**
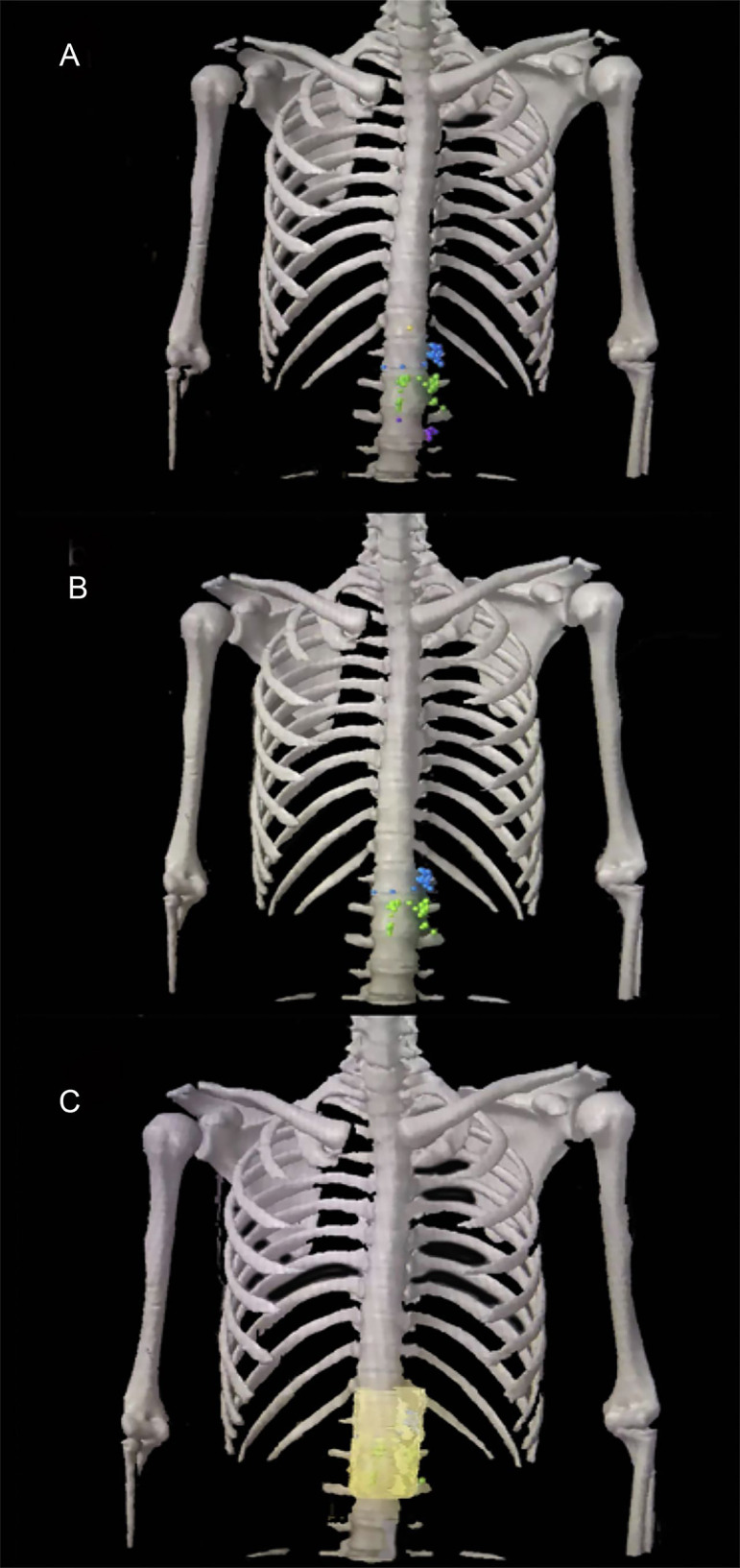
The para-aortic CTV coverage in the validation cohort. **(A)** PALN map in the validation cohort. **(B)** Lymph node map of regions 16a2 and 16b1 in the validation cohort. **(C)** Coverage of the para-aortic CTV in the validation cohort. CTV, clinical target volume; PALN, para-aortic lymph node. Orange plot, metastatic lymph nodes in 16a1; blue plot, metastatic lymph nodes in 16a2; green plot, metastatic lymph nodes in 16b1; purple plot, metastatic lymph nodes in 16b2.

A total of 56 PALNs in the validation cohort were projected onto the standardized axial CT images (**Figures 4A, B**). Within the range from the superior border of the celiac trunk to the superior border of the inferior mesenteric artery, the mean distance from the center of the node to the aorta was 10 mm (range 5–20) on the left. Similarly, the average distance on the right was also 10 mm (range 6–23). The distance in front of the aorta is 9 mm (range 5–23) (**Table 4**). The expansion margins calculated from the study cohort were applied to the validation cohort, and 96% (n = 47/49) coverage of lymph nodes was obtained (**Figure 4C**).

**Table 4:** Distance from the aorta to the volumetric center of the PALN in LN16a2 and LN16b1 in the validation cohort.

Location of PALNs	Mean distance from the aorta (mm) (range)	The 90th percentile of the distances (mm)	The 95th percentile of the distances (mm)
LPA	10 (5–20)	12	13
RPA	10 (6–23)	15	20
APA	9 (5–23)	12	18

PALN, para-aortic lymph node; LPA, left para-aortic; RPA, right para-aortic; APA, anterior para-aortic.

### 3.7 Prognostic impact of distant metastases in postoperative patients

To explore the reasonability of the CTV including the para-aortic region, the survival profile of BTC patients with different distant metastases was evaluated. The median OS of all relapsed patients was 18.9 months (IQR 9.8–32.95). The patients with PALN recurrence had a better prognosis (hazard ratio (HR) = 0.219, 95% confidence interval (CI) 0.067–0.711, P = 0.006) than patients with distant organ metastases (**Supplementary Figure 9A**). There was no significant difference in survival between the patients with PALN recurrence and those with intrahepatic metastases (HR = 1.183, 95% CI 0.539–2.594, P = 0.675) (**Supplementary Figure 9B**). However, the OS in the patients with intrahepatic recurrence was significantly better than that in the patients with distant organ metastases (HR = 0.367, 95% CI 0.142–0.948, P = 0.031) (**Supplementary Figure 9C**). Adjuvant radiotherapy was recommended for the para-aortic region according to the postoperative recurrence patterns of BTC in many previous retrospective studies. However, those studies did not evaluate the necessity of the CTV including the para-aortic region on the perspective of survival. Although PALN metastases belong to distant metastases, BTC patients with PALN metastases had a better survival compared with other organ metastases. Therefore, it was reasonable to incorporate the para-aortic region as the CTV for adjuvant radiotherapy.

## 4 Discussion

Lymphatic metastasis is a common metastasis form in biliary malignant tumors. In this study, we summarized and compared the pattern of LNMs in different cohorts and found that the involvement rate of the para-aortic region was the highest. The PALNs were mainly concentrated in the 16a2 and 16b1 regions. Based on the distribution of PALNs, we defined a more accurate para-aortic CTV to provide a reference for the CTV delineation of adjuvant radiotherapy. Meanwhile, we evaluated the impact of PALN recurrence on survival, adding evidence for the rationality of adjuvant radiotherapy of the para-aortic region.

The patients in the study cohort were those newly diagnosed BTC patients with lymph node metastasis, whereas the patients in the validation cohort were those patients with lymph node recurrence after radical resection. The reason is as follows (1): The surgical resection might affect the regularity of postoperative lymphatic drainage. Moreover, antitumor treatment will also affect the pattern of the regional failure. If the pattern of the lymphatic metastasis of BTC patients is biased, it is hard to draw an objective and accurate CTV. Therefore, the newly diagnosed BTC patients with metastasis were enrolled in the study cohort. (2) The purpose of adjuvant radiotherapy is to prevent local recurrence after surgery. The lymph node distribution of postoperative recurrence can be used to examine whether the current study-proposed para-aortic CTV could effectively cover the PALN metastasis. Therefore, the patients in the validation cohort were those postoperative patients with lymph node recurrence.

We collected all positive lymph nodes and found that the involvement rates of LNM in the 16b1 and 16a2 areas were significantly higher than those in the 16a1 or other regional lymphatic drainage areas. Regional lymph node areas, including 7, 8, and 12, showed moderate involvement rates. The involvement rates of other lymphatic drainage areas in the abdomen were less than 5%. The subgroup analysis showed that the involvement rate of LNM in each region was slightly different in different tumor sites. In fact, many previous studies have summarized the pattern of recurrence of BTCs after surgery (16). Jung et al. (17) analyzed the local recurrence patterns of 36 cases of distal eCCA after pancreatoduodenectomy. They found that the involvement rate of the para-aortic region was 16.1%, that of the superior mesenteric artery area was 16.1%, and that of the portal vein area was 14.5%. Another study (18) summarized the recurrence rate of 145 patients with CCA and found that the recurrence rates of the portal vein and retroperitoneal lymph nodes were 16.3% and 17.4%, respectively. However, in this study, we only summarized the distribution of lymph nodes. The distribution trend of PALNs in the validation cohort was similar to that in the study cohort. The proportion of PALNs was still higher than that of the first-station regional lymphatic drainage area, and it was mainly concentrated in 16b1 and 16a2. This further confirmed that the para-aortic region was a high-risk area for LNM. The involvement rate of 16b1 was greater than that of 16a2 in each cohort, whereas the involvement rates of 16a1 and 16b2 were both lower. However, the involvement rate of 16b1 in patients with postoperative metastasis was much higher than that in patients with their first diagnosis, and the rates of 16a2 and 16b2 were also slightly higher, which may be related to the changes in the anatomical structure caused by the resection.

At present, surgical resection in patients with BTC with PALN metastasis remains controversial. The guidelines do not recommend the resection of the primary site when with PALN metastasis. The PALN, as the final lymph node of the abdominal lymphatic system from the bile duct, is related to the poor prognosis of biliary malignancies (19, 20) and corresponds to M1 in the TNM classification. Many previous retrospective studies (16, 21, 22) suggested adjuvant radiotherapy for the para-aortic region based on the postoperative recurrence pattern of biliary malignancies. However, those studies did not evaluate the necessity of the CTV including the para-aortic region on the perspective of survival. In this study, postoperative patients with PALN recurrence had a better prognosis than patients with other distant organ recurrence. The prognosis of patients with PALN recurrence was different from that of patients with other organ recurrence. Therefore, it was reasonable to incorporate the para-aortic region as the CTV for adjuvant radiotherapy. Unfortunately, there were few patients only with regional lymph node recurrence, so we cannot compare the survival difference between them and the patients with PALN recurrence. Moreover, previous studies have found that the incidences of postoperative lymph node recurrence and microscopic venous infiltration are extremely high in patients with PALN metastasis (23). Undoubtedly, radiotherapy is the most effective means to control regional recurrence (21, 24).

The term “para-aortic” is not truly defined in the radiotherapy of BTC. Early studies lacked a specific description of the boundary of the para-aortic CTV, which may lead to major inconsistencies in the boundary of the target region. Choi et al. (22) summarized the postoperative failure pattern of eCCA and found that most local recurrences occurred between the T10/11 interspace and the L1/2 interspace. They believed that 16a2 (11.3%) should be included in the treatment volume, and 16a1 (1.4%), 16b1 (1.4%), and 16b2 (0%) should be omitted to reduce RT-related toxicity. Hughes et al. (21) believed that the para-aortic CTV range of adjuvant radiotherapy for distal CCA should be from the inferior border of T10 to the inferior border of L2. Similarly, Mallick et al. (16) also suggested that the para-aortic CTV should extend from at least 1 cm of the cranial or portal lymph node station to the L2 level. Compared with their results, the CTV calculated in this study had a slightly lower inferior border because of the low involvement rate of LNM in 16a1. There is almost no metastasis in the 16a1 region in eCCA. The superior of L3 was recommended as the caudal border of the CTV. This seems to be lower than the results from previous studies. The difference may be related to the tendency of PALNs to distribute downward in the patient cohort. Regarding the expansion margins for CTV delineation, Mallick et al. (16) believed that the para-aortic CTV should be constructed by expanding the aorta by a margin of 3–3.5 cm on the right, 2–2.5 cm in the front, 1–1.5 cm on the left, and 0.2 cm in the back. Compared with our results, the CTV created according to Mallick may be considered too generous. Their proposed margin may lead to unnecessary irradiation and a higher renal dose, especially in the right of the aorta. We obtained a relatively small para-aortic CTV, which can still provide satisfactory coverage of the PALNs. At the same time, this asymmetric para-aortic CTV could better protect the surrounding normal tissue.

This study has some potential limitations. First, the metastatic lymph nodes were only confirmed on imaging and not by pathology. Indeed, CT, MRI, and PET-CT have high sensitivity and specificity in identifying LNM based on different sequences. Second, no recommendations are offered for the para-aortic CTV according to different tumor locations. However, regardless of subgroups, the involvement rates of 16b1 and 16a2 were greater than those of 16a1 and 16b2. In addition, the low sample size leading to low statistical power is non-neglectable. Finally, fixed points were used to represent metastatic lymph nodes based on the assumption that the metastatic lymph nodes expand symmetrically. If the purpose is to cover micrometastasis, the lymph node center will represent the area to be covered. This method has been used in the previously published literature (15, 25, 26).

## 5 Conclusions

In this study, the distributions of LNM in patients initially diagnosed and in patients with postoperative recurrence were summarized, and the involvement rates of LNM in para-aortic regions 16a2 and 16b1 were the highest. Based on the distribution of PALNs, a new para-aortic CTV was defined to construct a more accurate target volume for adjuvant radiotherapy in BTC.

## Data availability statement

The raw data supporting the conclusions of this article will be made available by the authors, without undue reservation.

## Ethics statement

The studies involving human participants were reviewed and approved by the ethics committee (EC) of the Zhongnan Hospital of Wuhan University. Written informed consent for participation was not required for this study in accordance with the national legislation and the institutional requirements.

## Author contributions

FZ and XL designed the study. XL and JP drafted the manuscript. HW, LY, and HX participated in the data collection. JD, WW, and LX coordinated, edited, and finalized the drafting of the manuscript. All authors contributed to the article and approved the submitted version.

## Funding

This study was funded by the National Natural Science Foundation of China (NSFC 81800563) and the Leading Discipline Construction Support Project of Oncology in Zhongnan Hospital of Wuhan University (XKJS202005).

## Acknowledgments

The authors would like to express their sincere thanks to the National Natural Science Foundation of China and Zhongnan Hospital of Wuhan University.

## Conflict of interest

The authors declare that this research was conducted in the absence of any commercial or financial relationships that could be construed as a potential conflict of interest.

## Publisher’s note

All claims expressed in this article are solely those of the authors and do not necessarily represent those of their affiliated organizations, or those of the publisher, the editors and the reviewers. Any product that may be evaluated in this article, or claim that may be made by its manufacturer, is not guaranteed or endorsed by the publisher.
