# Supplementary material for: Recommendations of the clinical target volume for the para-aortic region based on the patterns of lymph node metastasis in patients with biliary tract cancer
Source: Front Oncol. 2022 Nov 3;12:893509. doi: 10.3389/fonc.2022.893509 (PMC9668861; doi:10.3389/fonc.2022.893509)
Supplement: Supplementary file 1 [file DataSheet_1.docx]

Supplementary Materials

Supplementary figures


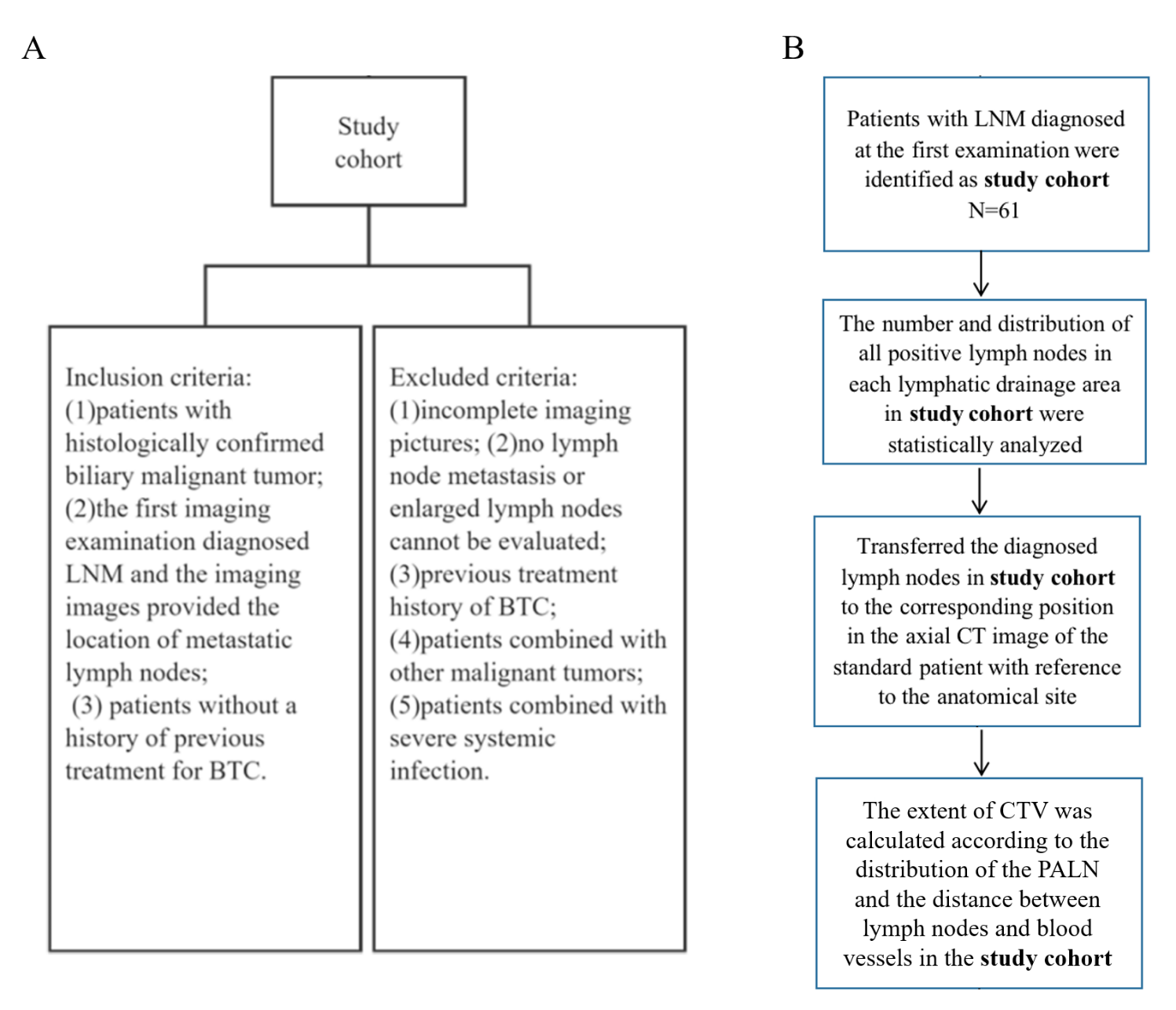


**Supplementary figure 1. Patient selection criteria and the study flow chart in the study cohort.** A. Inclusion and exclusion criteria for patients in the study cohort; B. The study flow chart in the study cohort. LNM, lymph node metastasis; BTC, biliary tract cancer; PALN, para-aortic lymph node


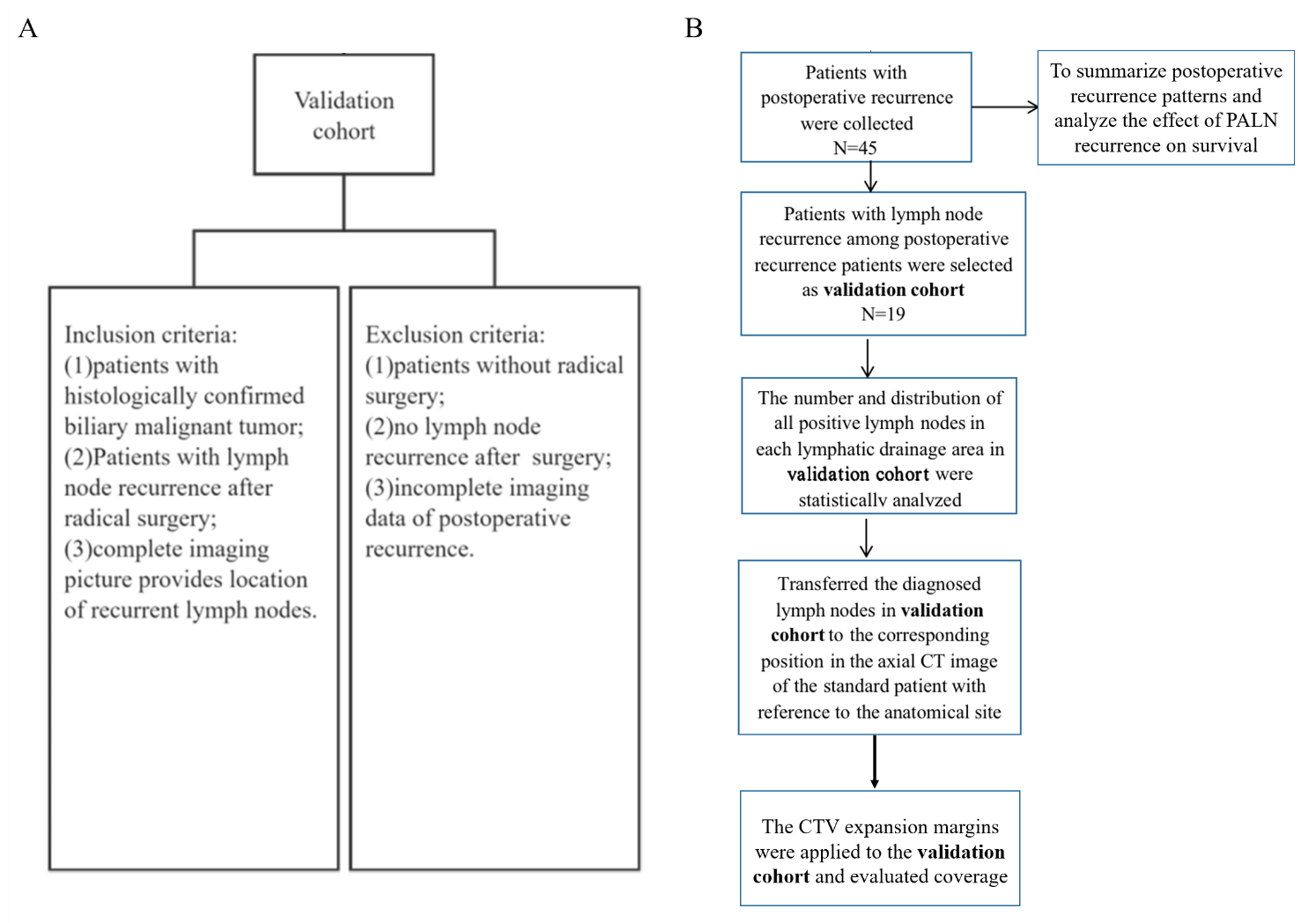


**Supplementary figure 2. Patient selection criteria and the study flow chart in the validation cohort.** A. Inclusion and exclusion criteria for patients in the validation cohort; B. The study design flow chart in the validation cohort. LNM, lymph node metastasis. BTC, biliary tract cancer; PALN, para-aortic lymph node


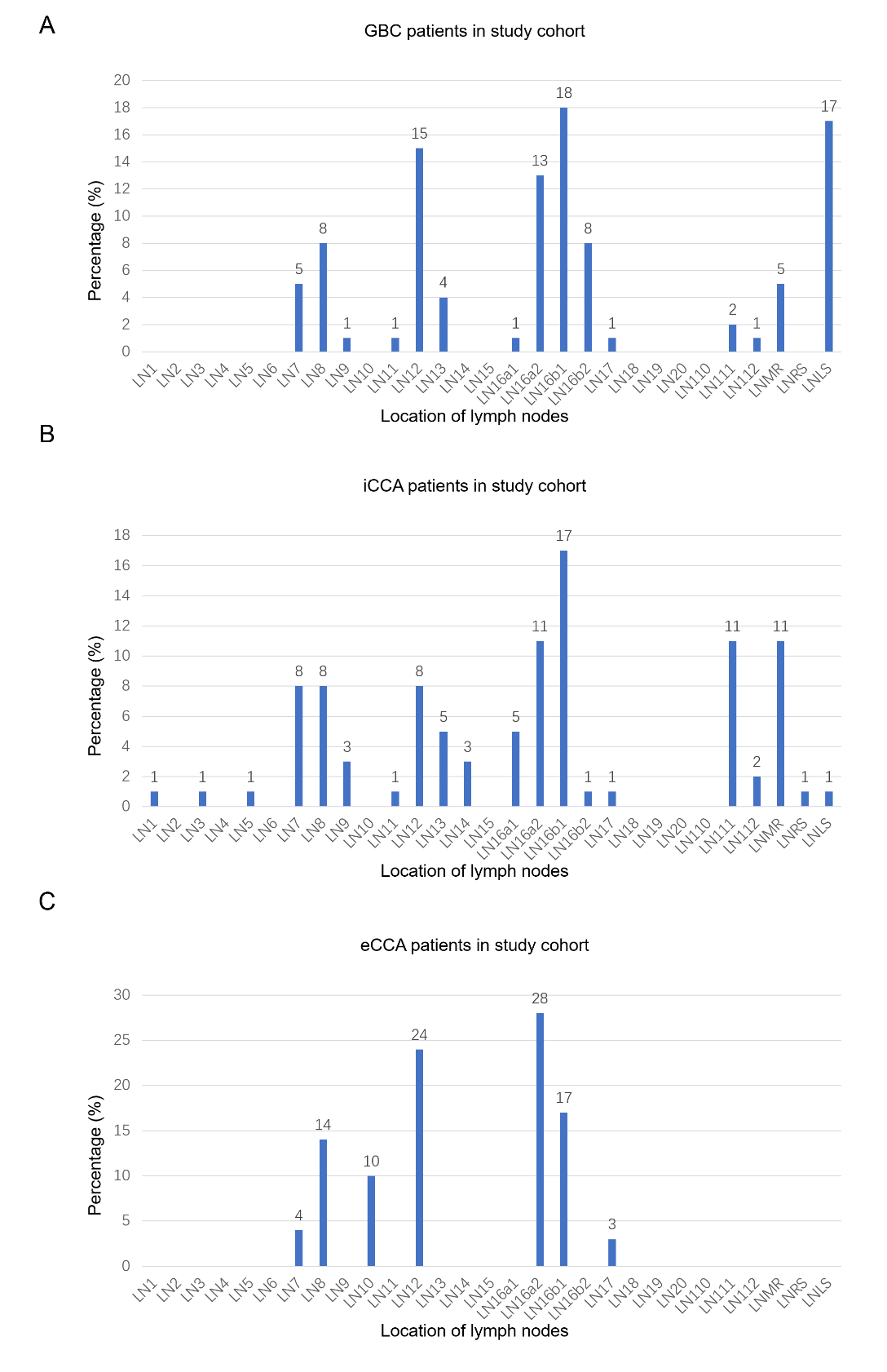


**Supplementary figure 3. Location of positive lymph nodes in the subtype of BTC patients in study cohort.** A. Percentage of positive lymph nodes in different locations in the GBC patients; B. Percentage of positive lymph nodes in different locations in the iCCA patients; C. Percentage of positive lymph nodes in different locations in the eCCA patients. BTCs, biliary tract cancer; iCCA, intrahepatic cholangiocarcinoma; GBC, gallbladder carcinoma; eCCA, extrahepatic cholangiocarcinoma; LN, lymph node; LNMR, mediastinal lymph node; LNRS, right supraclavicular lymph node; LNLS, left supraclavicular lymph node.


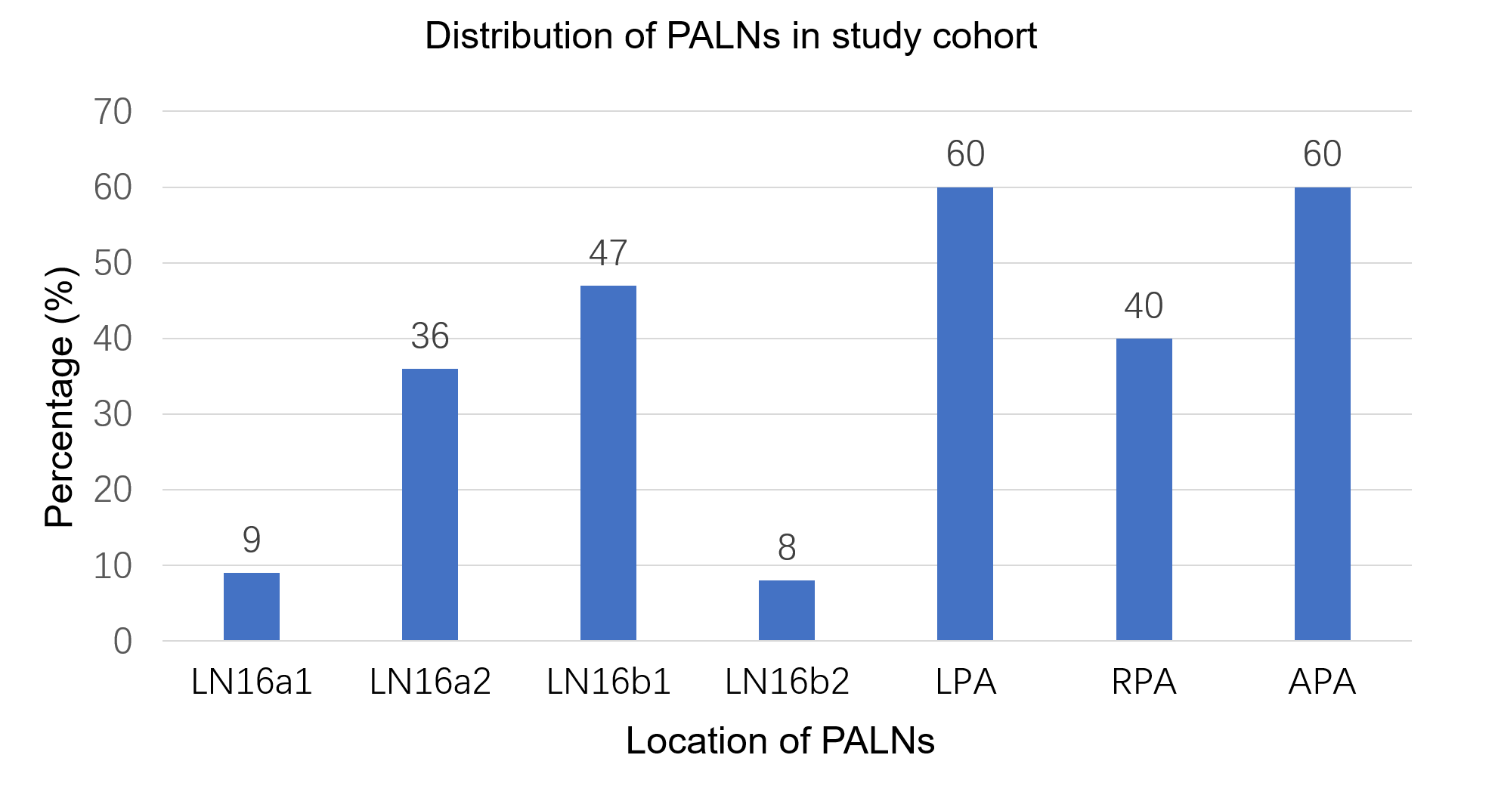


**Supplementary figure 4. Distribution of PALNs in study cohort.** LN, lymph node; PALN, para-aortic lymph node; LPA, left para-aortic; RPA, right para-aortic; APA, anterior para-aortic


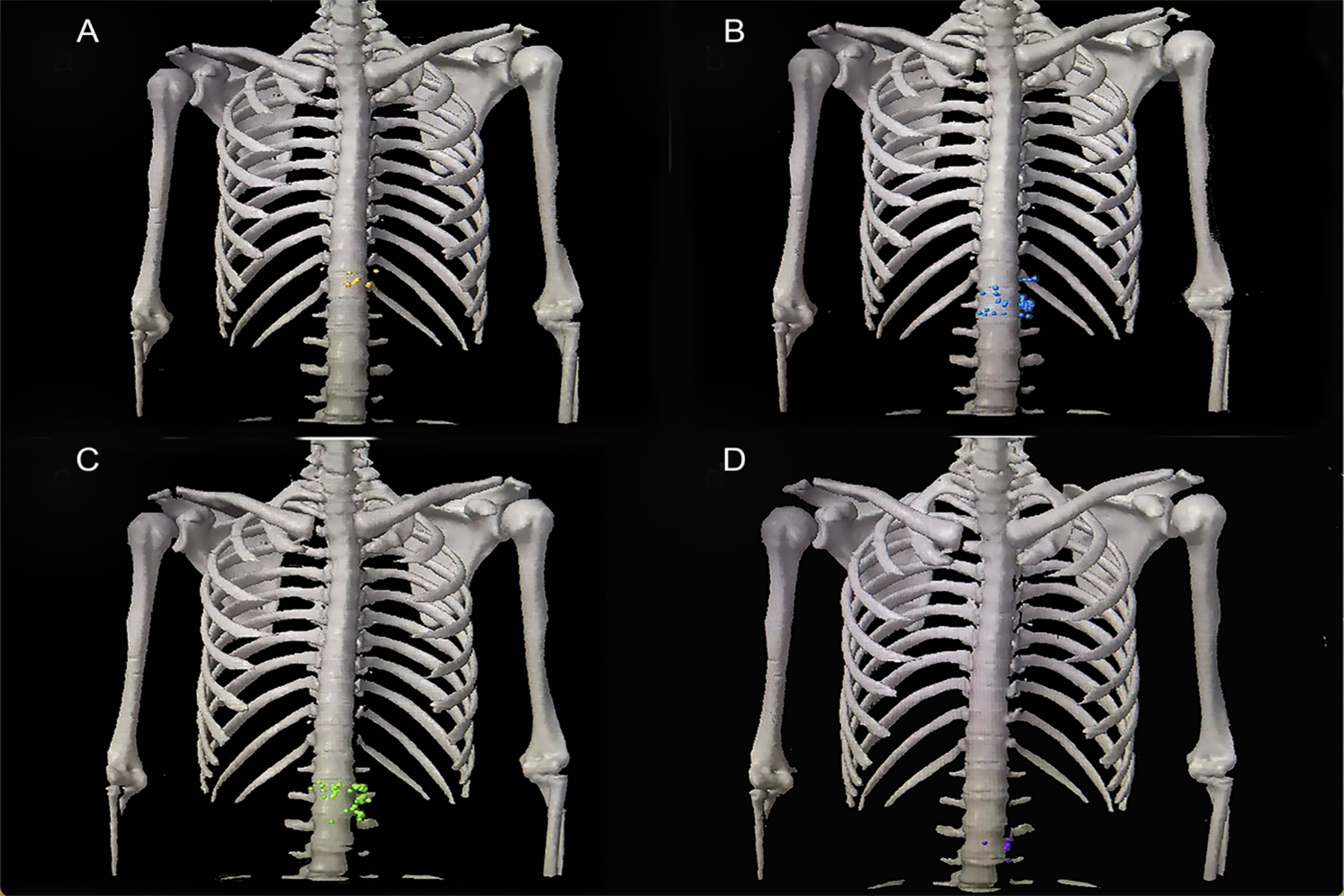


**Supplementary figure 5. PALN map in the study cohort.** A. LN map of region 16a1; B. LN map of region 16a2; C. LN map of region 16b1; D. LN map of region 16b2. PALN, para-aortic lymph node; LN, lymph node; Orange plot, metastatic lymph nodes in 16a1; Blue plot, metastatic lymph nodes in 16a2; Green plot, metastatic lymph nodes in 16b1; Purple plot, metastatic lymph nodes in 16b2.


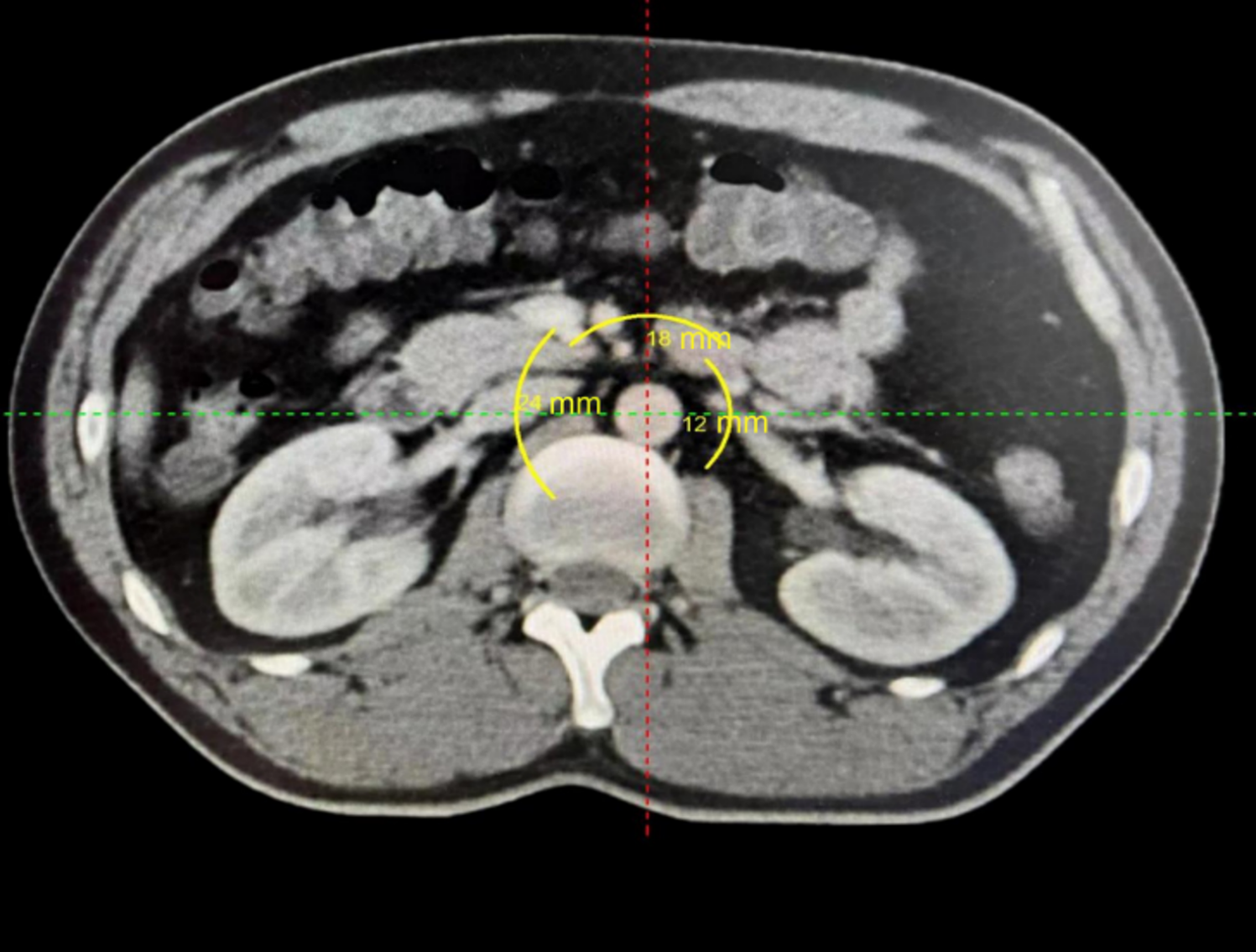


**Supplementary figure 6. Schematic diagram of expansion margins in the study cohort.** Expansion margins from the aorta of 18mm in the front, 12 mm on the left, and 24 mm on the right.


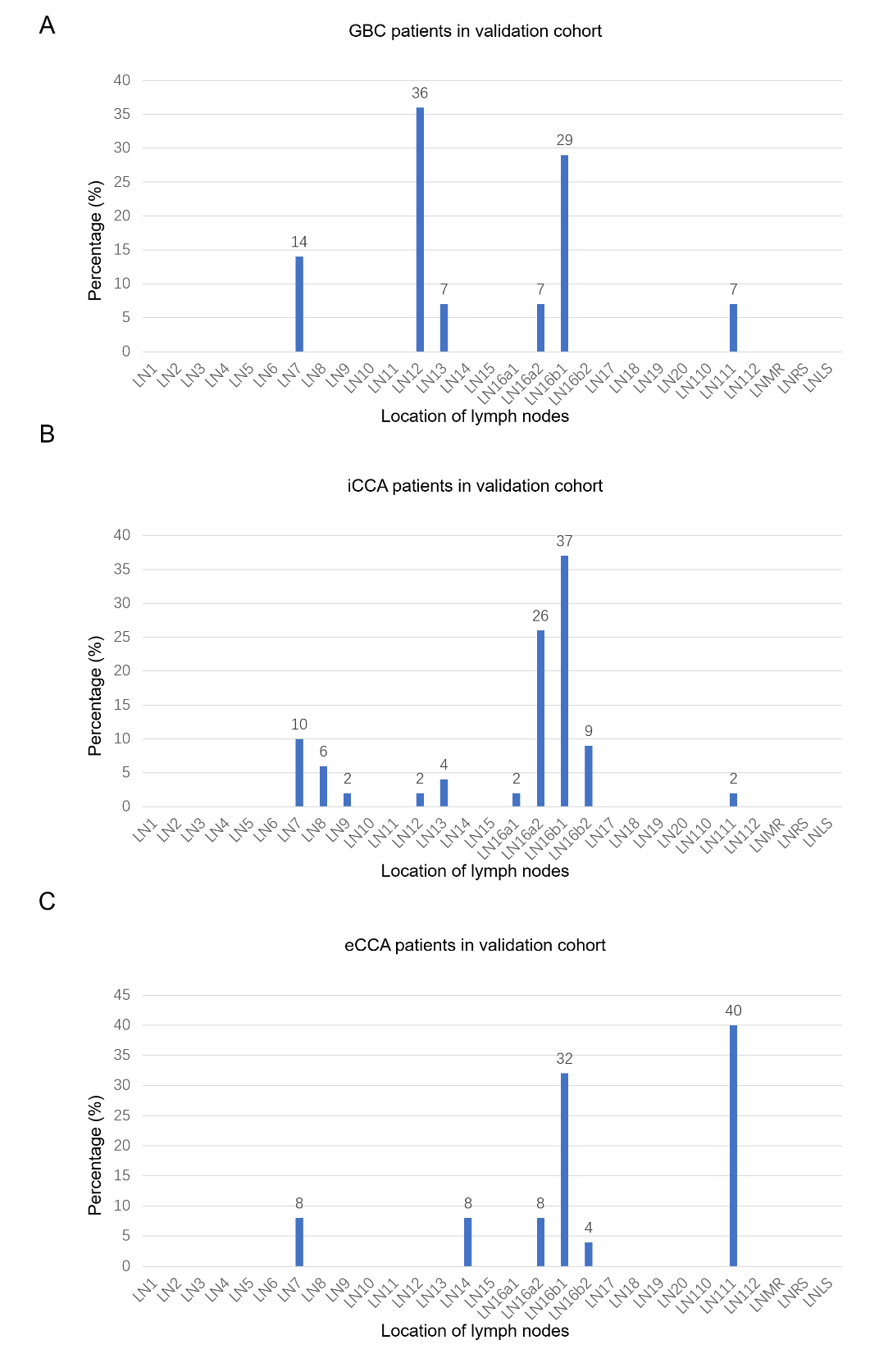


**Supplementary figure 7. Location of positive lymph nodes in the subtype of BTC patients in the validation cohort.** A. Percentage of positive lymph nodes in different locations in the GBC patients; B. Percentage of positive lymph nodes in different locations in the iCCA patients; C. Percentage of positive lymph nodes in different locations in the eCCA patients. BTCs, biliary tract cancer; iCCA, intrahepatic cholangiocarcinoma; GBC, gallbladder carcinoma; eCCA, extrahepatic cholangiocarcinoma; LN, lymph node; LNMR, mediastinal lymph node; LNRS, right supraclavicular lymph node; LNLS, left supraclavicular lymph node.


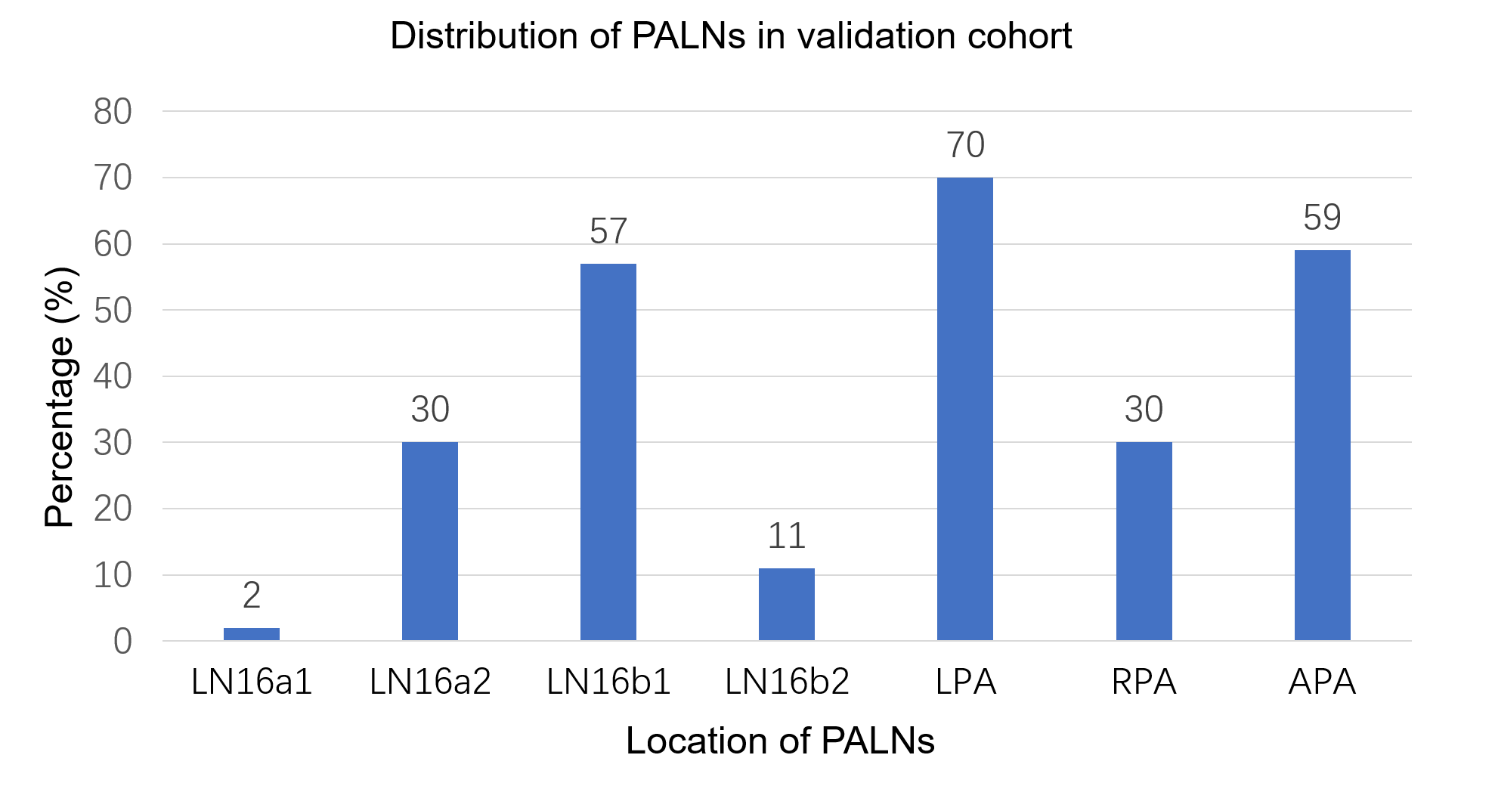


**Supplementary figure 8. Distribution of PALNs in the validation cohorts.** LN, lymph node; PALN, para-aortic lymph node; LPA, left para-aortic; RPA, right para-aortic; APA, anterior para-aortic


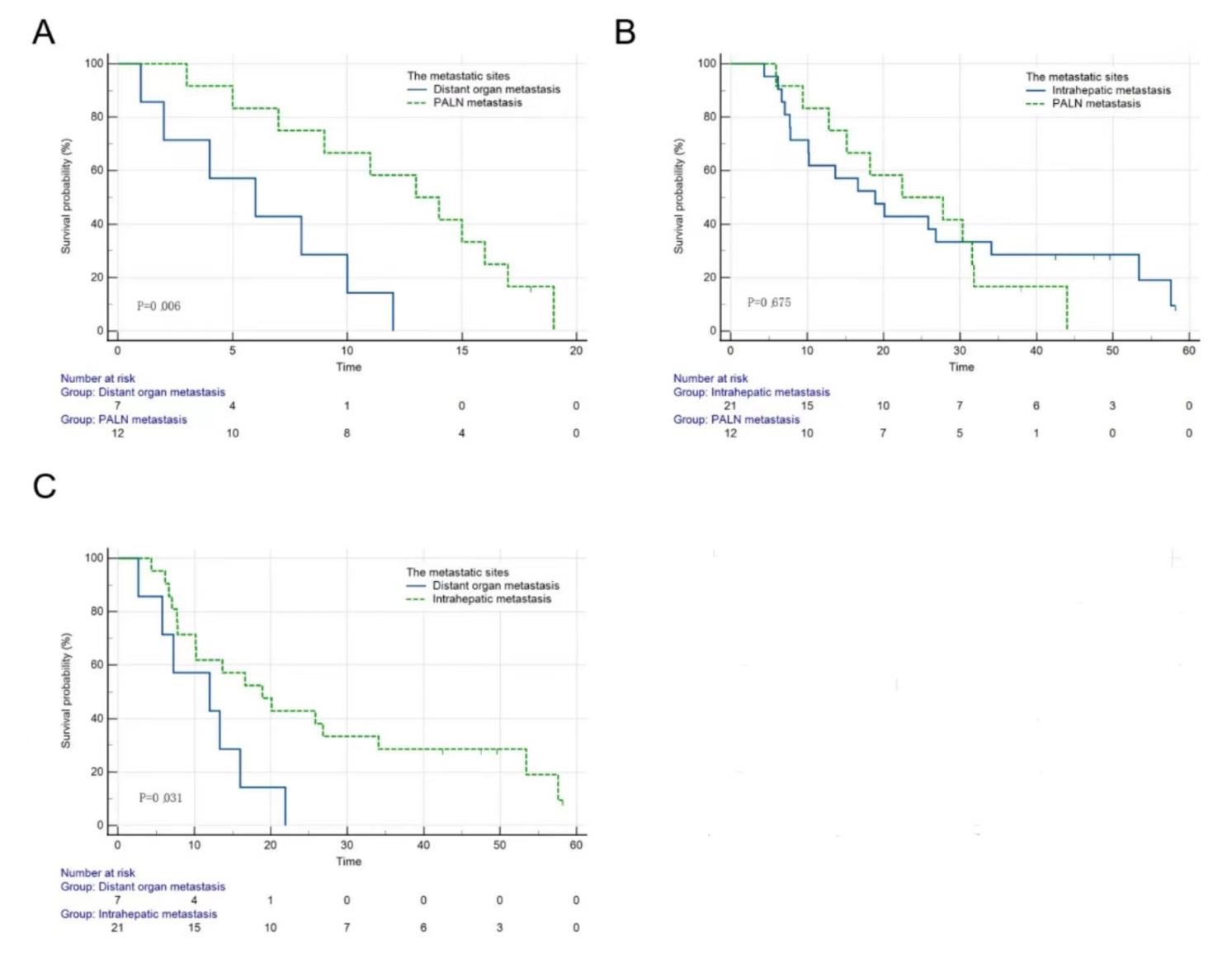


**Supplementary figure 9. Comparison of the survival profile in BTC patients with different sites of metastases in postoperative patients.** A. Comparison of the survival profile between BTC patients with PALN metastasis (n=12)and distant organ metastasis(n=7); B. Comparison of the survival profile in BTC patients with PALN metastasis (n=12) and intrahepatic metastasis (n=21); C. Comparison of the survival profile between BTC patients with intrahepatic metastasis (n=21) and those with distant organ metastasis (n=7). BTCs, biliary tract cancer; PALN, para-aortic lymph node.

**Supplementary table 1**. Characteristics of patients with postoperative recurrence

| **Variable** | **Value** |
| --- | --- |
| Age(y), median(IQR) | 58(51.5-65) |
| Sex |  |
| Male | 22(49%) |
| Female | 23(51%) |
| Tumor Location |  |
| Gallbladder | 12(26%) |
| Intrahepatic bile duct | 18(40%) |
| Extrahepatic bile duct | 15(34%) |
| Tumor size (cm), median(IQR) | 3.5(2.0-5.75) |
| Pathologic T stage |  |
| T1-2 | 23(51%) |
| T3-4 | 22(49%) |
| Pathologic N stage |  |
| N- | 20(45%) |
| N+ | 11(24%) |
| Nx | 14(31%) |
| Pathological type |  |
| Adenocarcinoma | 43(95%) |
| Other types | 2(5%) |
| Recurrence location |  |
| Lymph node ^a^ | 19(42%)* |
| Intrahepatic^b^ | 21(47%) |
| Distant organ^c^ | 7(16%)* |

IQR, interquartile range; a, refers to including lymph node recurrence, regardless of whether it is combined with other sites recurrence, 14 of these patients had PALN recurrence; b, only intrahepatic metastasis occurred, not accompanied by other sites recurrence; c, refers to distant organ recurrence, regardless of whether it is combined with other sites recurrence; *, two patients had both lymph node and distant organ recurrence.
